# Repositioning of Hypoglycemic Drug Linagliptin for Cancer Treatment

**DOI:** 10.3389/fphar.2020.00187

**Published:** 2020-03-03

**Authors:** Yong Li, Yiqun Li, Dengke Li, Kaiming Li, Zhengyang Quan, Ziyi Wang, Zhenxiao Sun

**Affiliations:** School of Life Sciences, Beijing University of Chinese Medicine, Beijing, China

**Keywords:** the oncology genome atlas project, drug repositioning, multi-target anti-tumor drug screening, molecular docking, cell proliferation and apoptosis, xenograft tumor mice, gene regulatory network

## Abstract

**Background:**

Drug repositioning, development of new uses for marketed drugs, is an effective way to discover new antitumor compounds. In this study, we used a new method, filtering compounds *via* molecular docking to find key targets combination.

**Methods:**

The data of gene expression in cancer and normal tissues of colorectal, breast, and liver cancer were obtained from The Cancer Genome Atlas Project (TCGA). The key targets combination was obtained from the protein-protein interaction network (PPI network) and the correlation analysis of the targets. Molecular docking was used to reposition the drugs which were obtained from DrugBank. MTT proliferation assay and animal experiments were used to verify the activity of candidate compounds. Flow cytometric analysis of proliferation, cell cycle and apoptosis, slice analysis, gene regulatory network, and Western blot were performed to elucidate the mechanism of drug action.

**Results:**

CDK1 and AURKB were identified as a pair of key targets by the analysis of different expression gene from TCGA. Three compounds, linagliptin, mupirocin, and tobramycin, from 12 computationally predicted compounds, were verified to inhibit cell viability in HCT116 (colorectal), MCF7 (breast), and HepG2 (liver) cancer cells. Linagliptin, a hypoglycemic drug, was proved to inhibit cell proliferation by cell cycle arrest and induce apoptosis in HCT116 cells, and suppress tumor growth in nude mice bearing HCT116 cells. Linagliptin reduced the tumor size and decreased the expression of Ki67, a nuclear protein expressed in all proliferative cells. Gene regulatory network and Western blot analysis suggested that linagliptin inhibited tumor cell proliferation and promoted cell apoptosis through suppressing the expression and phosphorylation of Rb, plus down-regulating the expression of Pro-caspase3 and Bcl-2, respectively.

**Conclusion:**

The combination of key targets based on the protein-protein interaction network that were built by the different gene expression of TCGA data to reposition the marketed drugs turned out to be a new approach to discover new antitumor drugs. Hypoglycemic drug linagliptin could potentially lead to novel therapeutics for the treatment of tumors, especially for colorectal cancer. Gene regulatory network is a valuable method for predicting and explaining the mechanism of drugs action.

## Introduction

World Health Organization (WHO) study showed that tumor morbidity and mortality were still rising gradually in recent years (Siegel et al., 2017). Therefore, the market for anti-tumor drugs is huge. It is getting difficult to find new drugs using the traditional methods, which drives the cost of new drugs research and development increasing and the success rate decreasing year by year (Yoonjeong et al., 2018). Drug repositioning, development of new uses for marketed drugs, has been proved to be an effective way to develop new anti-tumor drugs (Reddy and Zhang, 2013).

The Cancer Genome Atlas Project (TCGA, https://cancergenome.nih.gov/) was an analytical study of tumor molecular biology which was operated by the National Cancer Institute (NCI) and the National Human Genome Research Institute (NHGRI) in 2006 (Tomczak et al., 2015). TCGA uses a large-scale sequencing-based genomic analysis technology to clarify the molecular mechanism of cancer through extensive cooperation, which improves scientific understanding of the molecular biology of cancer, and enhance the ability to diagnose and prevent cancer (Weinstein et al., 2013).

In present study, we analyzed the different gene expression in cancer and their adjacent normal tissues of colorectal, breast, and liver cancer, which obtained from TCGA. The key targets combination was obtained according to the protein-protein interaction network (PPI network) and the correlation of targets. Molecular docking was used to reposition the drugs which were obtained from DrugBank (Wishart et al., 2006; Wishart et al., 2008) and 12 compounds were obtained. Three of the 12 compounds were tested on tumor cell viability *in vitro*, and the traditional hypoglycemic drug linagliptin was chosen for further study. The effects of linagliptin on tumor cell division, cell cycle, and apoptosis was determined by flow cytometric analysis. The gene regulatory network was used to predict and interpret the mechanism of linagliptin, and the changes of key targets expression in the possible signal pathways were analyzed by Western blot.

## Materials and Methods

### The Combination of Key Targets

The data of gene expression of colorectal cancer, breast cancer, liver cancer tissue, and normal tissues were obtained from TCGA. The gene expression between tumor tissues and normal tissues were compared by using Bioconductor. The genes whose |LogFC| is more than 2 and *p*-value is less than 0.05 were defined as the different gene.

The protein-protein interaction networks of different gene were constructed by STRING10.0 (Szklarczyk et al., 2015). The information of PPI targets was obtained from STRING10.0, which include physical and functional association interaction. The interactions were evaluated by score. The higher the score, the more reliable of the protein interactions it is. We selected score which is more than 0.7 to ensure the reliability of data (Zheng et al., 2016). The parameters of network nodes were calculated by network analyzer, and we selected the degree centrality more than 10 to filter the key targets. We analyzed the expression and correlation of the key targets to combine the key targets.

### Virtue Screening

The structures of targets were obtained from PDB (http://www.rcsb.org) and were prepared by Discovery Studio 2.5 (DS2.5), and the ligand file were prepared in the same manner.

The active site of targets was defined by the original ligand. Then, Libdock, a method of semi-flexible molecular docking based on hot matching were used to screen the marketed drugs. Root mean square deviation (RMSD) were calculated to evaluate the preferences and the binding pockets. The information of the binding pockets was adjusted to make the RMSD score less than 2.0, which could perform a better binding mode between the original ligand and the proteins, and the Libdock score was recorded. The compounds with the score more than the threshold, which was 80% of the original ligand Libdock score, and the interaction pattern similar to the original ligand were selected as candidate compounds (Yong et al., 2016; Yong et al., 2018).

The Libdock scores of the selected compounds based on the different target were summed and sorted. The 3% of approved compounds before the score were selected with no antitumor activity reported, which were defined as potential compounds.

### Cell Lines and Reagents

The colorectal cancer cell lines, HCT 116, the liver cancer cell line, HepG2, and the breast cancer cell line, MCF7, were obtained from National Infrastructure of Cell Line Resource (NICLR) and cultured in RPMI1640 medium (Gibco, Invitrogen, USA) with 10% FBS (Vistech, SE100-011 Fetal Bovine Serum, Qualified, Uruguay), 100 U/ml penicillin (Gibco, Invitrogen, USA), and 100 μg/ml streptomycin (Gibco, Invitrogen, USA) in a humidified atmosphere of 5% CO_2_ at 37°C. Linagliptin was obtained from Macklin (Macklin, Shanghai, China), mupirocin from Yuanye (Yuanye, Shanghai, China), and tobramycin from Hefeng (Hefeng, Shanghai, China), respectively.

### MTT Proliferation Assay

The MTT reagent (Amresco, USA) can be metabolized by active cells to formazan, an insoluble purple dye that can be measured in a spectrophotometer, and was used according to the manufacturer's instructions. Cells were seeded in quadruplicates in 96-well plates (1,600 per well). After 24-h adhesion time, the cells were treated with the compounds chosen, and anticancer product norcantharidin (NCTD) (Sigma, USA) in positive group (Zimu and Zhenxiao, 2015). 0, 24, 48, and 72 h later, 100 μl of MTT reagent were added to each well and the cells were again incubated for 4 h at 37°C and 5% CO_2_. After incubation and 10 minutes shaking, the absorbance was measured on a Genios Microplate Reader (Spectra Max190). All experiments were performed in triplicates.

### Flow Cytometric Analysis of Proliferation, Cell Cycle, and Apoptosis

Flow cytometric assay was used for proliferation, cell cycle and apoptosis assays. Brieﬂy, for cell proliferation determination, equal numbers of HCT116 cells (4.8×10^4^) were seeded in the per well of 6-well plate and cell adherent growth, washed twice with PBS, added CFSE with the final concentration 5 mM (Kaiming et al., 2018). The cells were then incubated for 10 min in dark. 4~5 ml serum-containing pre-cooled medium was added to stop the reaction on ice for 5 min and incubated with different concentration of linagliptin (0, 100 μM) for 24 h. All experiments were performed in triplicates.

For cell cycle determination (Chen et al., 2016), equal numbers of HCT116 cells (4.8×10^4^) were seeded in 6-well plate per well and incubated with different concentration of linagliptin (0, 50, 100 μM) for 48 h. The cells were washed with PBS and then collected, and fixed in 70% ice cold ethanol, and storage at 4˚C for at least 8 h. Cells were then washed with PBS twice, and centrifuged for 5 min at 1000 rpm and aspirate the supernatant, and resuspended cells in 0.5 ml of PI/RNase staining buffer (BD Pharmingen) for 60 min at 4˚C, and DNA content was immediately analyzed using ﬂow cytometry analysis. All experiments were performed in triplecates.

For cell apoptosis determination (Ziyi et al., 2019), equal numbers of HCT116 cells (4.8×10^4^) were seeded in 6-well plate per well and incubated with different concentration of linagliptin (0, 100 μM) for 48 h. Cells were collected and cell apoptosis was detected according to the instructions of the apoptosis assay kit (KeyGen Biotech, Jiangsu, China). All experiments were performed in triplicates.

### Animal Experiments

All animal experiments strictly adhered to local regulations as well as LAWER (Laboratory Animal Welfare Ethics Review) guidelines (Andersen and Winter, 2017; Herrmann and Flecknell, 2018), and were approved by the local authorities before initiation.

1×10^6^ tumor cells in 100 μl of PBS were injected into the back of BALB/c-nu mice. When the tumors reached an average volume of 40 mm^3^, mice were treated as follows for 14 days. The group treated with PBS was designated as the control. The group treated with cyclophosphamide (CTX) (20 mg/kg, three times per week) was designated as the positive control. The tumor volume (tv) and the body weight of BALB/c-nu mice were monitored three times per week. The tv was calculated using the following formula: tv= ab^2^/2, where a is the length of the tumor, and b is the width. The tumors were separated and weight after mice euthanizing.

The treatment regimens were as follows:

Tumor-bearing control group: 200 μl PBS buffer i.p. daily. (n = 6)
Tumor-bearing positive group: 30 mg/kg body weight (cyclophosphamide) i.p. daily, dissolved in 200 μl PBS in the subcutaneous experiment. (n = 5)
Linagliptin low dose group: 50 mg/kg body weight p.o. daily, dissolved in 200 μl PBS. (n = 5)
Linagliptin high dose group: 500 mg/kg body weight p.o. daily, dissolved in 200 μl PBS. (n = 5)

### Immunohistochemistry Analysis

Formalin-fixed tumor tissues were embedded in paraffin and cut into 4-µm sections. The sections were deparaffinized by heating in Tris-EDTA (pH 9.0) buffer. The slides were treated with 3% hydrogen peroxide to block endogenous peroxidase activity and then incubated with goat serum for 30 min. Next, the slides were incubated overnight at 4˚C with anti-Ki67 antibody (Abcam ab16667, 1:400 diluted in TBST) and the rest of the steps were carried out according to the manufacturer's instructions using SP (rabbit IgG)-POD Kit (Solarbio, Beijing, CN). Immunohistochemically stained slides for Ki67 were scanned using microscope with a 40× objective. Ten representative images selected from two groups were then analyzed using Image J, which segmented cells with positive and negative nuclei. The percentage of the area containing positive cells was calculated as the brown area (positively stained cells) divided by the sum of brown and blue areas (negatively stained cells). The software interpretation was manually verified by visual inspection of the digital images to ensure accuracy.

### Gene Regulatory Network Analysis for Mechanism of Linagliptin

Gene regulatory network, a Boolean network model, was constructed based on the Python 2.7. Numpy, Pandas, networkx, matplotlib, and BooleanNet were used to construct a Boolean network model for colorectal cancer gene regulation (Albert et al., 2008; van der Walt et al., 2011; Fuchs et al., 2015; Kadiyala and Kumar, 2017). The colorectal cancer gene interactions in this model were derived from the KEGG database (Kanehisa and Goto, 1999; Kanehisa et al., 2002)

According to the activation or inhibition of the target of linagliptin, the nodes were set to “open” or “closed”, and the other parameters are the same as default until the network state is stable, and the expression of colorectal cancer gene after the action of linagliptin is analyzed (Kauffman et al., 2003; Cheng and Qi, 2010; Yue et al., 2018). Gene enrichment analysis was used to analyze the different genes by ClueGo, which is a plugin in Cytoscape (Shannon et al., 2003; Bindea et al., 2009).

### Western Blot

Total protein from HCT116 cells were extracted using standard methods (Feng et al., 2018) and protein concentrations were determined by BCA protein assay kit (Thermo scientific, USA). A total of 40 µg of protein were separated by SDS-PAGE. Separated proteins were transferred onto a NC (nitrocellulose) membrane at 200 mA for 2 h. After transfer, membranes were blocked with 5% BSA in 1X PBST (phosphate-buffered saline with 0.1% Tween 20) at room temperature for 4 h. The membrane was probed with p53 (Zsbio, CN), Bcl-2 (Abcam, USA), Pro-caspase3 (Cell Signaling Technology, USA), Rb (Cell Signaling Technology, USA), pRb^s780^ (Cell Signaling Technology, USA), pRb^s807/811^ (Cell Signaling Technology, USA) or β-actin primary antibodies (Zsbio, CN) overnight at 4˚C and then secondary antibody at RT for 1 h according to manufacturer's instructions.

### Statistical Analysis

Student *t-*test was used to compare means. All analyses were two tailed, and *p* < 0.05 was considered statistically significant (^*^, *p* < 0.05; ^**^, *p* < 0.01; ^***^, *p* < 0.001).

## Results

### The Combination of Key Targets

PPI networks were constructed based on the different gene expression between colorectal cancer, breast cancer, liver cancer tissues, and normal tissues adjacent to cancer. All 10 nodes were obtained from protein-protein interaction analysis of 113 differential gene nodes in the protein interaction network between liver cancer tissue and normal tissues adjacent to cancer, which were defined as the key targets. Seventy-seven nodes were considered as the key targets in the PPI network of breast cancer, and 215 nodes were considered as the key targets in the PPI network of colorectal cancer.

Six targets were obtained from the intersections of the key targets, which were AURKB, BIRC5, CCNB2, CDC20, CCNB1, and CDK1. According to the preliminary screening results, it was found that the combination of double targets for drug screening is better than the combination of single target, triple targets, and all targets. Finally, CDK1 and AURKB were selected as the combination of the key targets according to the expression and correlation of the key targets.

### Screening of Novel Drug Compounds for CDK1 and AURKB

**Figure 1:**
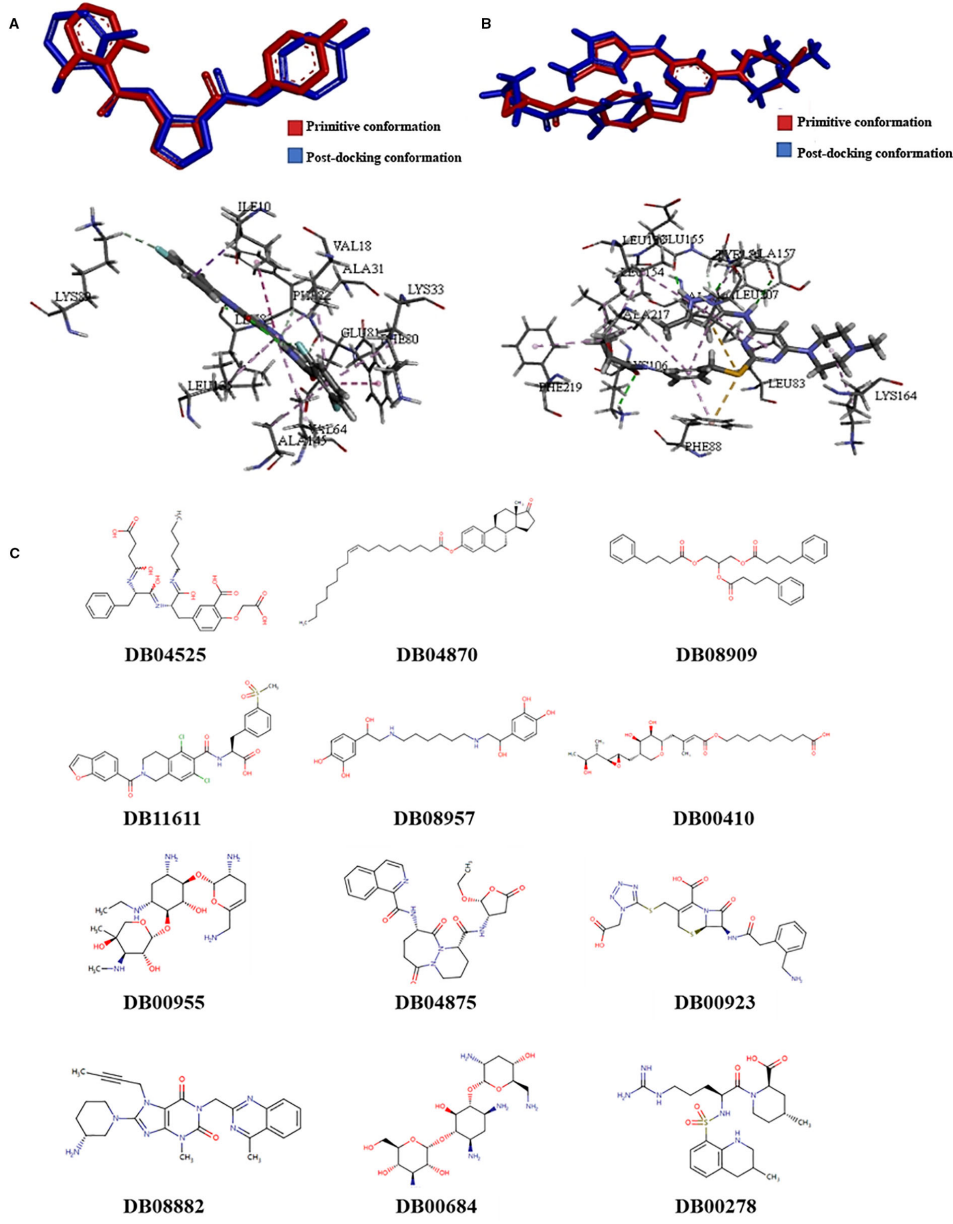
The results of screening of novel drug compounds for CDK1 and AURKB **(A)**: up: The comparison of conformations of the initial ligand before and after docking with CDK1; below: The interactions between initial ligand and CDK1; **(B)**: up: The comparison of conformations of the initial ligand before and after docking with AURKB; below: the interactions between initial ligand and AURKB; **(C)** The structures of candidate compounds.

Molecular docking models were built based on the structures of CDK1 (PDB code: 5HQ0) and AUKRB (PDB code: 4AF3) (Elkins et al., 2012; Brown et al., 2015). Compounds which were considered as the inhibitors of CDK1, the Libscores should be more than 130.76 and the interaction pattern should be similar to the original ligand, which could have interactions with LYS33, LEU83, GLU51, and GLU81 (**Figure 1A**). Compounds which were considered as the inhibitors of AURKB, the Libscores should be more than 145.84 and the interaction pattern should be similar to the original ligand, which could have interactions with PHE88 (**Figure 1B**).

Twelve compounds were obtained from Drugbank, which were considered as the inhibitors of CDK1 and AURKB, which were 2-(carboxymethoxy)-5-[(2s)-2-({(2s)-2-[(3-carboxypropanoyl)amino]-3-phenylpropanoyl}amino)-3-oxo-3-(pentylamino)propyl]benzoic acid (DB04525), oleoyl estrone (DB04870), glycerol phenylbutyrate (DB08909), lifitegrast (DB11611), hexoprenaline (DB08957), mupirocin (DB00410), netilmicin (DB00955), pralnacasan (DB04875), ceforanide (DB00923), linagliptin (DB08882), tobramycin (DB00684), and argatroban (DB00278). The structures of compounds were showed in the **Figure 1**. Linagliptin, mupirocin, and tobramycin were selected for *in vitro* experiments.

### Candidates Compounds Inhibits Cell Viability in a Dose- and Time-Dependent Manner

**Figure 2:**
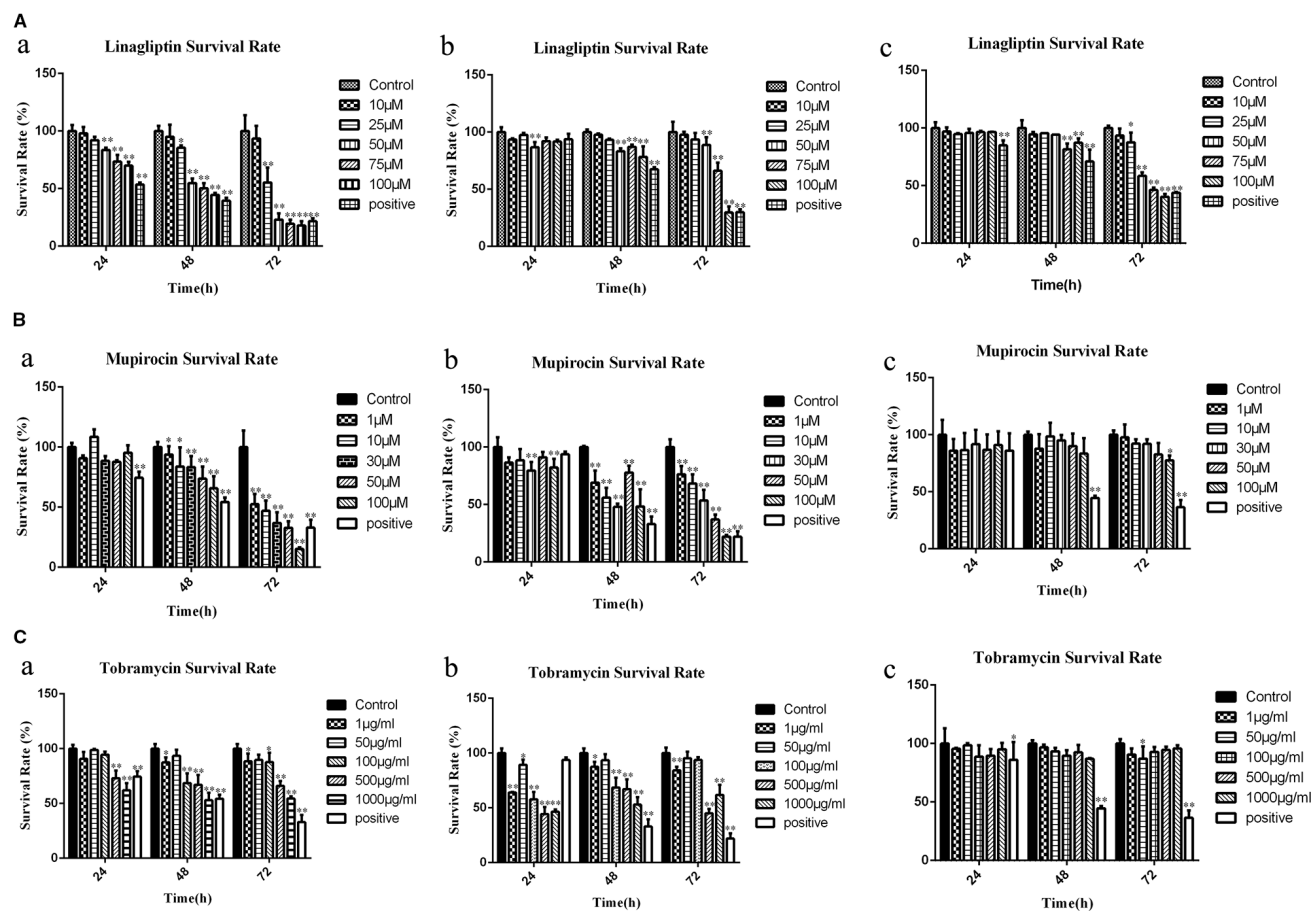
Candidate compounds inhibit cell viability in HCT116 cells, MCF7 cells, and HepG2 cells **(A)**: Linagliptin inhibits viability of HCT116 cells, MCF7 cells, and HepG2 cells, a showed linagliptin inhibits cell viability in HCT 116 cell line, b showed linagliptin inhibits cell viability in MCF7 cell line, c showed linagliptin inhibits cell viability in HepG2 cell line; **(B)**: Mupirocin inhibits cell viability in HCT116 cells, MCF7 cells, and HepG2 cells, a showed mupirocin inhibits cell viability in HCT 116 cell line, b showed mupirocin inhibits cell viability in MCF7 cell line, c showed mupirocin inhibits cell viability in HepG2 cell line; **(C)**: Tobramycin inhibits viability of HCT116 cells, MCF7 cells and HepG2 cells, a showed tobramycin inhibits cell viability in HCT 116 cell line, b showed tobramycin inhibits cell viability in MCF7, c showed tobramycin inhibits cell viability in HepG2 cell line.

To explore the cytotoxicity potential of linagliptin, mupirocin, and tobramycin against colon cancer, liver cancer and breast cancer, HCT116 cells, HepG2 cells, and MCF7 cells were treated with linagliptin, mupirocin, and tobramycin at various concentrations for 24, 48, and 72 h, which were shown in **Figure 2**.

Linagliptin inhibits HCT 116, MCF7, and HepG2 cell viability in dose- and time-dependent manner (**Figure 2A**). With the increase of linagliptin from 0 to 100 μM, cell viability decreased from 100 to 44.52% in HCT116 cells, from 100 to 78.34% in MCF 7 cells, and 100 to 87.22% in HepG2 cells at 48 h. Meanwhile, with the increase of time from 24 h to 72 h based on 100 μM linagliptin, cell viability decreased from 66.54 to 20.26% in HCT 116 cells, from 89.94 to 30.32% in MCF7 cells and 96.42 to 41.11% in HepG2 cells.

The inhibitory effect of mupirocin and tobramycin on HCT116 cells, HepG2 cells, and MCF7 cells were shown in **Figures 2B, C**. HCT 116 cells were sensitive to mupirocin and tobramycin, cell viability decreased from 100 to 65.95% with the increase of mupirocin from 0 to 100 μM and decreased from 100 to 54.35% with the increase of tobramycin from 0 to 1000 μg/ml at 48 h. Mupirocin inhibits the cell viability of MCF7 cells in a dose- and time-dependent manner, and tobramycin had the strongest inhibitory effect on MCF7 at 500 μg/ml after 48 h treatment. HepG2 cells were not dramatically sensitive to the candidate of compounds except linagliptin.

### Linagliptin Inhibits Cell Proliferation *In Vitro*

**Figure 3:**
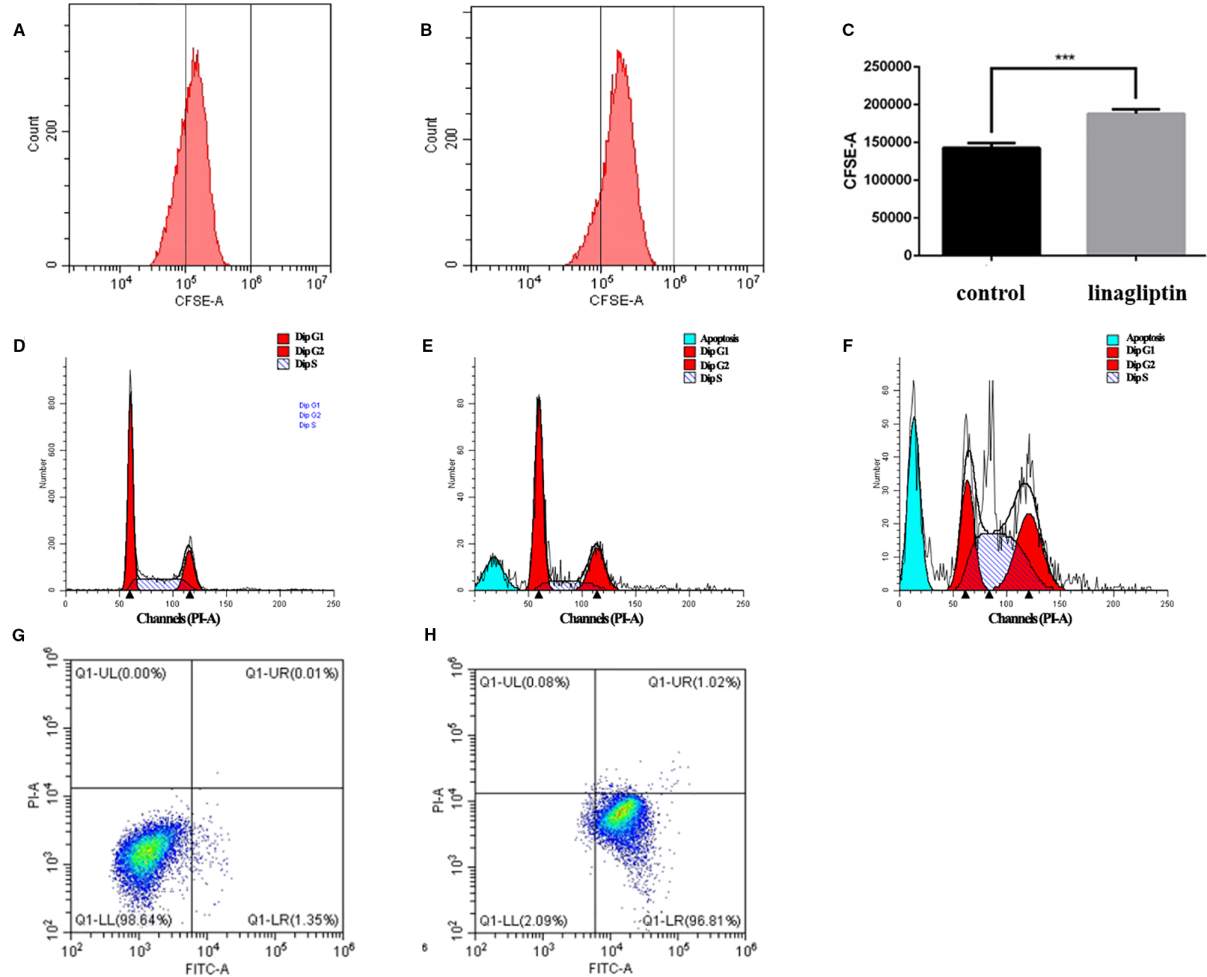
Linagliptin induces HCT 116 cell cycle arrest and apoptosis **(A)**: Control fluorescence intensity of CFSE; **(B)**: Fluorescence intensity of CFSE in linagliptin treated group (HCT 116 cells were treated with 100 μM linagliptin for 24 h); **(C)**: Statistical difference between control group and linagliptin treated group in A; D–F: Cell cycle analysis by flow cytometry. **(D)**: Cell cycle of control group; **(E)**: 50 μM linagliptin induced cell cycle arrest at 48 h; **(F)**: 100 μM linagliptin induced cell cycle arrest at 48 h; **(G, H)**: Apoptosis analysis by flow cytometry. **(G)**: Control group; **(H)**: 100 μM linagliptin induced HCT 116 cell apoptosis at 48 h.

In order to reveal the anti-tumor mechanism of linagliptin in HCT116 cells, we examined the effects of linagliptin on the distribution of cell proliferation by ﬂow cytometry analysis. As shown in the **Figures 3A–C**, the fluorescence intensity of HCT 116 cells significantly increase upon linagliptin treatment. Compared to control cells, linagliptin significantly inhibits cell proliferation, with the fluorescence intensity from 142550.8 ± 7959.9 to 191519.1 ± 11222.8, which shown linagliptin could inhibit HCT 116 cell proliferation significantly.

### Linagliptin Induces Cell Cycle Arrest at G2/M and S Phase

In order to investigate the anticancer mechanism which linagliptin inhibits HCT116 cells proliferation, we determined the effects of linagliptin on cell cycle distribution by ﬂow cytometry analysis. Linagliptin could induce cell cycle arrest at G2/M phase in low dose, and induce cell cycle arrest at G2/M and S phase in high dose (**Figures 3D–F**). Compared to control cells (**Figure 3D**), low dose (50 μM) linagliptin significantly increased percentage of G2/M phase from 19.97 ± 0.52 to 25.11 ± 0.77, which were shown in the **Figure 3E**, high dose (100 μM) linagliptin significantly increased percentage of G2/M phase from 19.97 ± 0.52 to 31.07 ± 0.56 and percentage of S phase from 20.26 ± 1.64 to 45.17 ± 1.56, which were shown in the **Figure 3F**.

### Linagliptin Induces Apoptosis *In Vitro*

We observed obvious apoptotic peaks in ﬂow cytometry analysis cell cycle. To further elucidate the mechanism of cell death induced by linagliptin in HCT116 cells, ﬂow cytometry analysis were used to further study the ability of linagliptin in inducing apoptosis in HCT116 cells. As shown in **Figure 3G**–**H**, we observed that the amount of Annexin V+/PI− (early apoptosis) stained cells were increased significantly upon linagliptin (100 μM). The rate of early and late apoptotic cells was quantified and depicted in **Figure 3G**–**H**.

### Linagliptin Reduces Tumor Growth in Human Colorectal Cancer Cell HCT116-Bearing Xenografted Mice

**Figure 4:**
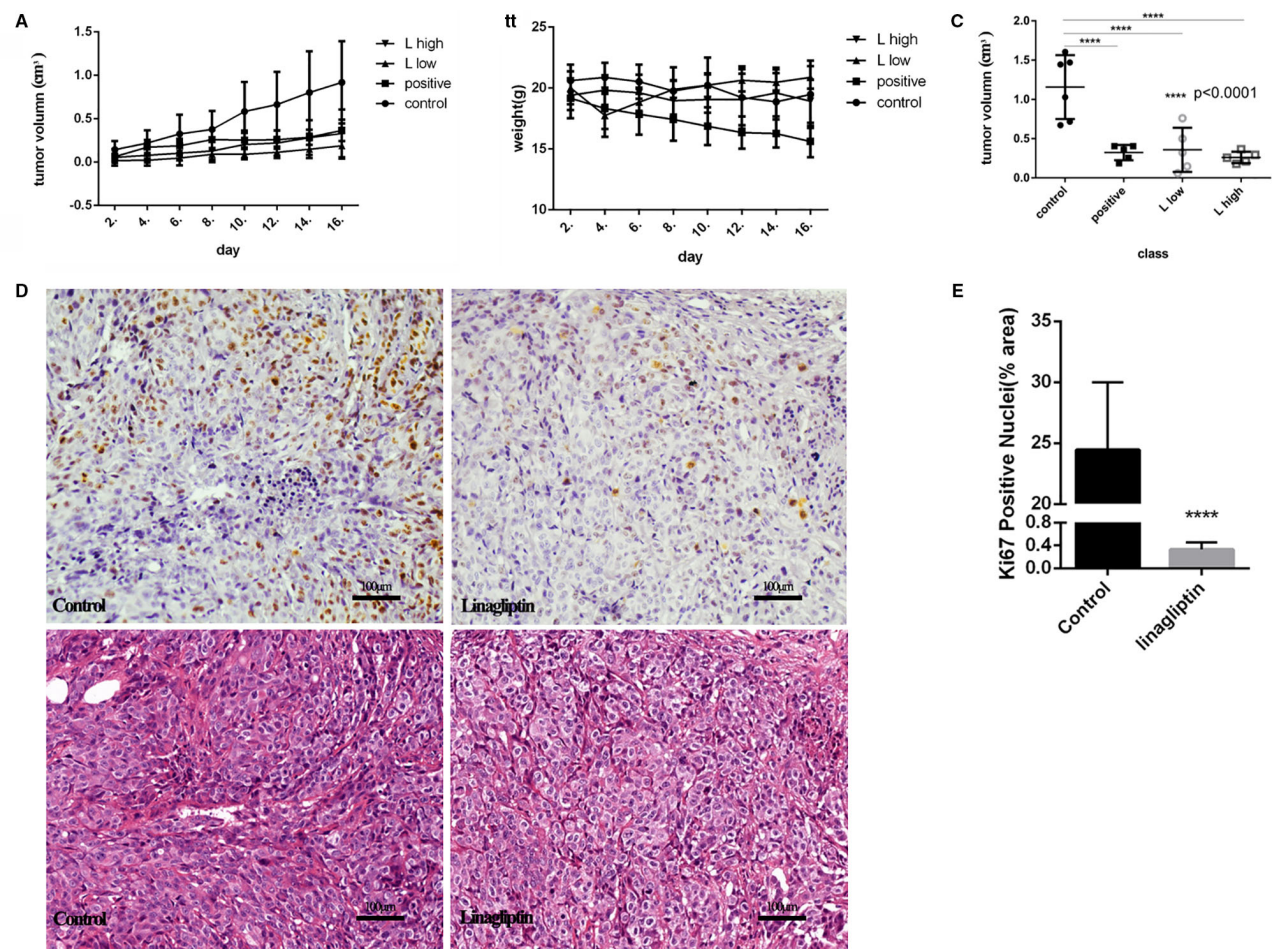
Linagliptin inhibits tumor growth in HCT116 xenograft nude mice **(A)**: Growth curve of tumor volume in nude mice; **(B)**: Effect of linagliptin and Cyclophosphamide treatment on body weight of nude mice; **(C)**: Change of tumor volume from control, cyclophosphamide, 50 mg/kg and 500 mg/kg linagliptin groups; **(D)**: Linagliptin reduced cell proliferation markers, Ki67, as determined by immunohistochemical staining (40×) and the graphs of H&E staining; **(E)**: The statistical analysis of Ki67 in control and linagliptin treated group showed in **(D)**.

To evaluate the antitumor effects of linagliptin *in vivo*, we examined the effects of low dose and high dose of linagliptin (50 mg/kg vs. 500 mg/kg) by oral gavage on tumor growth in human colorectal cancer cell HCT116-bearing xenografted mice. Cyclophosphamide was used as positive control. Both lower dose and higher dose of linagliptin inhibited the growth of HCT116 xenograft tumor dramatically (*p* < 0.05, **Figure 4A**). Tumor volume were reduced significantly by linagliptin (**Figure 4C**).

Compared to control group, the body weight of linagliptin treated mice did not change significantly, while the body weights of Cyclophosphamide treated group decreased significantly 2 weeks after injection (**Figure 4B**). In addition, no other adverse effects such as skin ulcerations or toxic death were observed in linagliptin groups. This result suggests that linagliptin may have less toxicity to mice compared to cyclophosphamide.

Ki67 is an important cell nuclear proliferation marker (Kun et al., 2017). The expression of Ki67 were markedly decreased in linagliptin treated groups compared with control group (**Figures 4D, E**), indicating that linagliptin effectively inhibited proliferation of HCT116 cells in human colon tumor-bearing xenografted mice.

### Linagliptin Inhibits Phosphorylation of Rb and Expressions of Bcl-2 and p53

**Figure 5:**
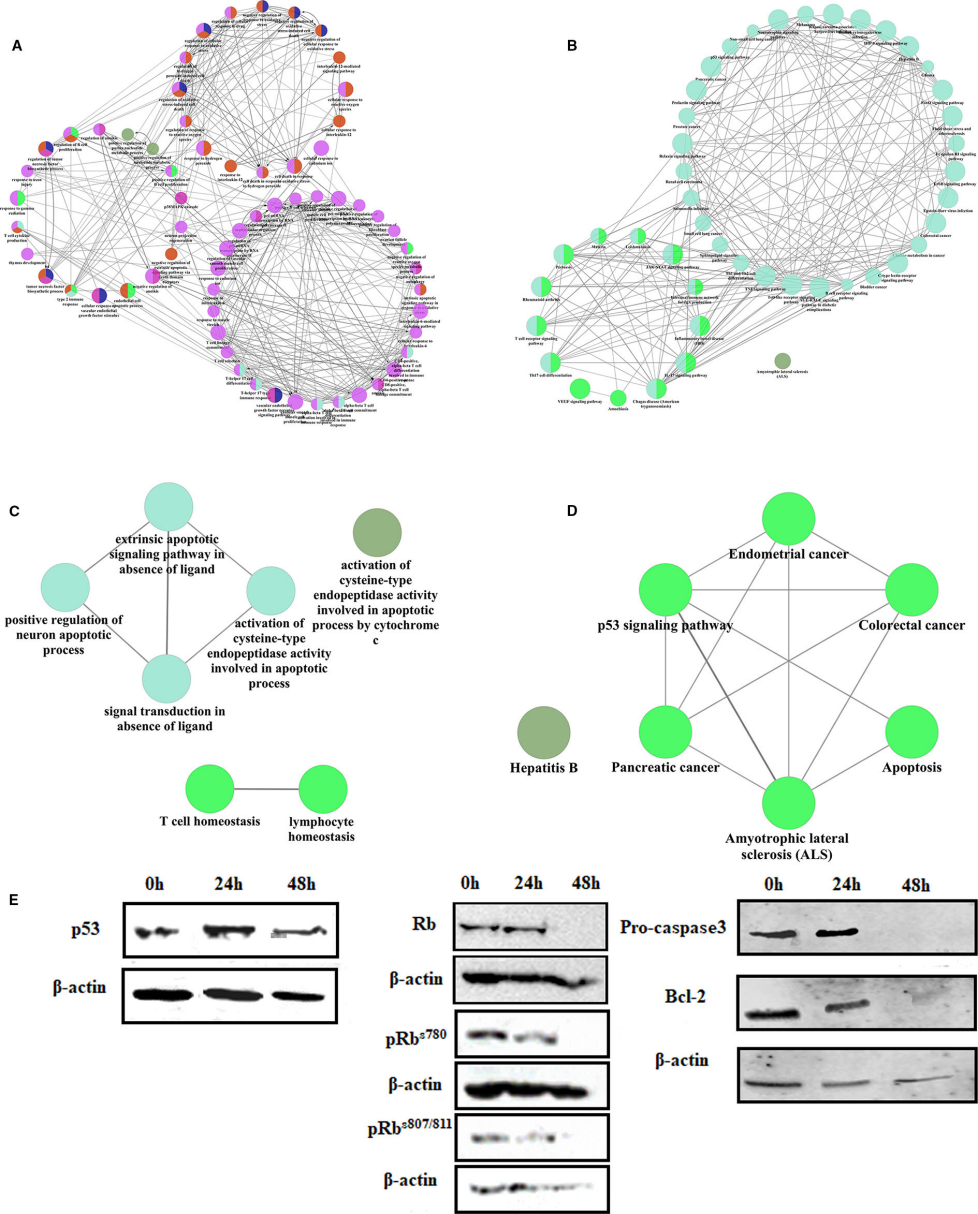
The mechanism of linagliptin inhibits tumor growth in HCT116 **(A)**: GO analysis of down-regulated genes from linagliptin effect gene regulatory network; **(B)**: KEGG analysis of down-regulated genes from linagliptin effect gene regulatory network; **(C)**: GO analysis of up-regulated genes from linagliptin effect gene regulatory network; **(D)**: KEGG analysis of up-regulated genes from linagliptin effect gene regulatory network; **(E)**: the expression levels of p53, Rb, pRb^s780^, pRb^s807/811^, Pro-caspase3 and Bcl-2.

Gene regulatory network were used to analyze the mechanism of linagliptin. There were 91 genes in the gene regulatory network of colorectal cancer, 40 genes were down-regulated and 21 genes were up-regulated which were shown in **Supplementary Material Table 1** (Δ > 0 means up regulated, and Δ < 0 means down regulated). Linagliptin could inhibit expression of *BCL2, MDM2,* and *STAT3*, which could inhibit JAK-STAT signaling pathway, and active the expression of *BAX, CASP9* and *P53*, which could active the pathway of apoptosis according to gene enrichment analysis, which were shown in the **Figures 5A–D**. Linagliptin could inhibits the proliferation of colorectal cancer cells and promotes apoptosis of colorectal cancer cells by acting on the above pathways and functions.

To confirm this result, the expression levels of key proteins such as p53, Bcl-2, Rb, and phosphorylation of Rb were examined by Western blot. As shown in **Figure 5E**, Rb, pRb^s780^, and pRb^s807/811^ were all significantly down-regulated by linagliptin at 48 h. The expression of p53 were significantly up-regulated by linagliptin at 24 h and 48 h. The expression of Bcl-2 and Pro-caspase3, which is related to cell apoptosis, were significantly down-regulated by linagliptin at 48 h.

## Discussion

The combination of key targets, CDK1 and AURKB, was obtained based on the different expression genes from TCGA, which were used to operate molecular docking. AURKB is a highly conserved serine-threonine protein kinase, which belongs to the Aurora family, and plays an important role in the regulation of mitosis (Lucenaaraujo et al., 2011). AURKB was detected over-expressed in pleomorphic gliomas, malignant mesothelioma, and hematological malignancies (Hall et al., 2009). Meanwhile, AURKB also over-expresses in colorectal cancer, liver cancer, and breast cancer according to the data from TCGA in this study. CDK1 could combined with cyclin A, cyclin B1, cyclin B2, and cyclin B3, which play an important role in the cell cycle (Chen et al., 2006). CDK1 could phosphorylate a variety of substrate proteins, such as histones H1, laminin, and Rb. Analyzing the expression and function of CDK1 and AURKB, the selected compounds could block cell proliferation and inhibit the growth of tumor. Then, we inferred the hypoglycemic drug linagliptin could potentially lead to novel therapeutics for the treatment of tumor, especially in colorectal cancer.

Linagliptin could inhibit cell viability by inducing cell cycle arrest at G2/M and S phase to block cell proliferation in HCT 116. Linagliptin could inhibit tumor growth, with the decreasing expression of Ki67 in colorectal cancer bearing xenografted mice, which is the clinically most relevant model to examine the effects of a compound on the development of tumor. Linagliptin was also detected to induce apoptosis in HCT 116 colorectal tumor cells.

Based on the result of gene regulatory network in systemic pharmacology, linagliptin could induce the decreasing of *JAK, MDM2, BCL2,* and induce the increasing of *BAX, CASP9* to inhibit JAK-STAT signal pathway, active p53 signal pathway and promote apoptosis pathway. On the basis of the results of molecular docking, the gene regulatory network and the result of Western blot, we propose that the main mode of action of linagliptin to inhibit cell proliferation and promote cell apoptosis is *via* the inhibition of the phosphorylation of Rb and the expression of Bcl-2, Pro-caspase3. Inhibition of JAK-STAT signal pathway and activation of p53 signal pathway may be induced by inhibition of CDK1 complex by linagliptin, which may be the main reason for the decrease of expression and phosphorylation of Rb according to the results of molecular docking (Johnson et al., 2016). The latest report showed linagliptin could inhibit hepatocellular carcinoma cells though suppressing protein ADORA3 and induce cell apoptosis at G2/M phase with increase in caspase3 levels (Ayoub et al., 2018), which supports our study results.

This study tried to mine target combinations from gene expression profiles and use the combination of key targets to reposition marketed drugs for discovering new anti-tumor drugs. Bioinformatics and molecular biology experiments were combined to explore the molecular mechanism of linagliptin. Target fishing combined with gene regulatory network was provided to reveal molecular mechanism of drug, which will be useful in systemic pharmacology in the future.

## Data Availability Statement

All datasets generated for this study are included in the article/**Supplementary Material**.

## Ethics Statement

The animal study was reviewed and approved by Laboratory Animals of Beijing University of Chinese Medicine. 

## Author Contributions

YoL filtered the candidate compounds in computer. YoL, YiL, ZQ, and ZW treated the animals and injected the stimulus. YoL, KL, and DL performed *in vitro* assays. YoL, YiL, and ZS analyzed and interpreted data set. YoL, DL, and ZS delineated the study. ZS received grants and provided essential reagents. YoL and ZS wrote, revised, and edited the manuscript. All authors read and approved the final version of the manuscript.

## Funding

This article was funded by the National Natural Science Foundation of China (81473418).

## Conflict of Interest

The authors declare that the research was conducted in the absence of any commercial or financial relationships that could be construed as a potential conflict of interest.
