# Supplementary material for: Repositioning of Hypoglycemic Drug Linagliptin for Cancer Treatment
Source: Front Pharmacol. 2020 Mar 3;11:187. doi: 10.3389/fphar.2020.00187 (PMC7062795; doi:10.3389/fphar.2020.00187)

The figures below were original ones for the Fig.5E, specific protein expression using Western blot analysis in the paper titled “Repositioning of hypoglycemic drug linagliptin for cancer treatment”. The selected lanes were in the red box, the expression of proteins such as p53, Rb, pRb^s780^, pRb^s807/811^, Pro-caspase3, Bcl-2 were in the above box and β-actin control in the red box below. The number of target bands in the new figure is the same as the old figure, and we re-added the bands of the internal reference, which is different with the old figure.


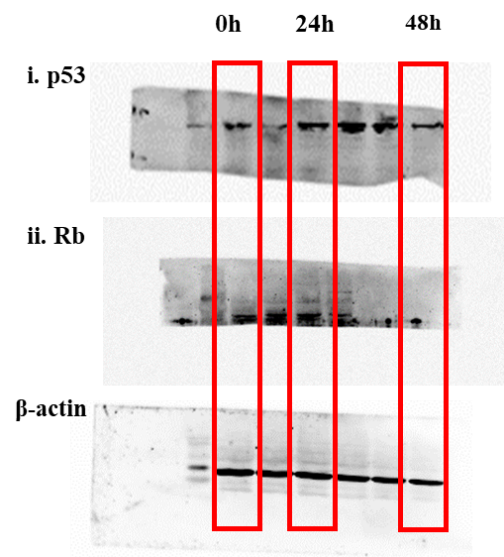


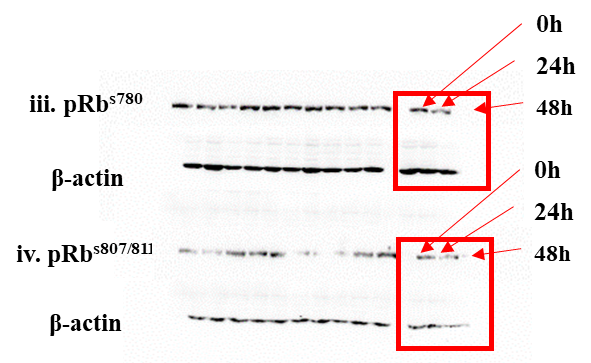


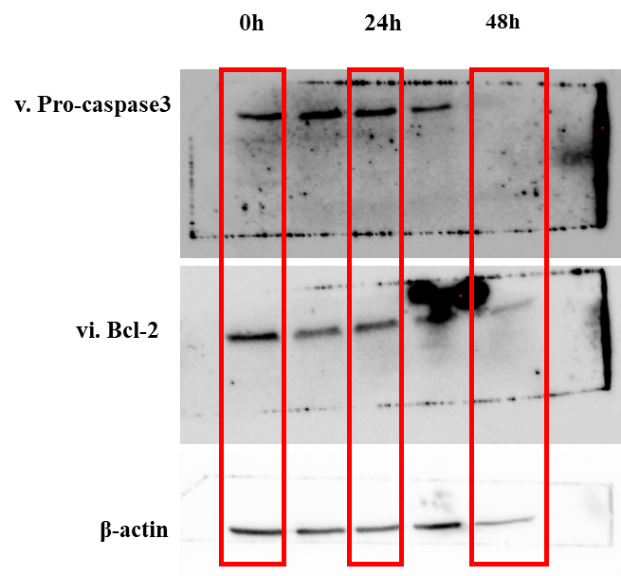

Supplement: Supplementary file 1 [file DataSheet_1.zip › 496241 original Western Blot images/origin figure/original images for Fig.5.docx]
