# Supplementary figures and images for: Repositioning of Hypoglycemic Drug Linagliptin for Cancer Treatment
Source: Front Pharmacol. 2020 Mar 3;11:187. doi: 10.3389/fphar.2020.00187 (PMC7062795; doi:10.3389/fphar.2020.00187)

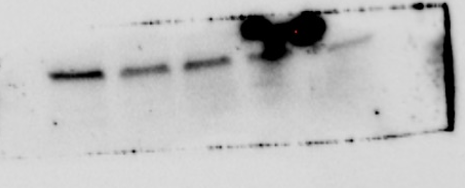

Supplement: Supplementary file 1 [file DataSheet_1.zip › 496241 original Western Blot images/origin figure/Bcl-2.tif]

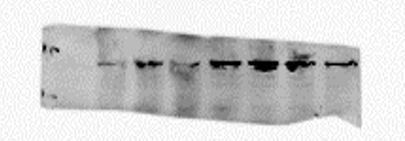

Supplement: Supplementary file 1 [file DataSheet_1.zip › 496241 original Western Blot images/origin figure/p53.tif]

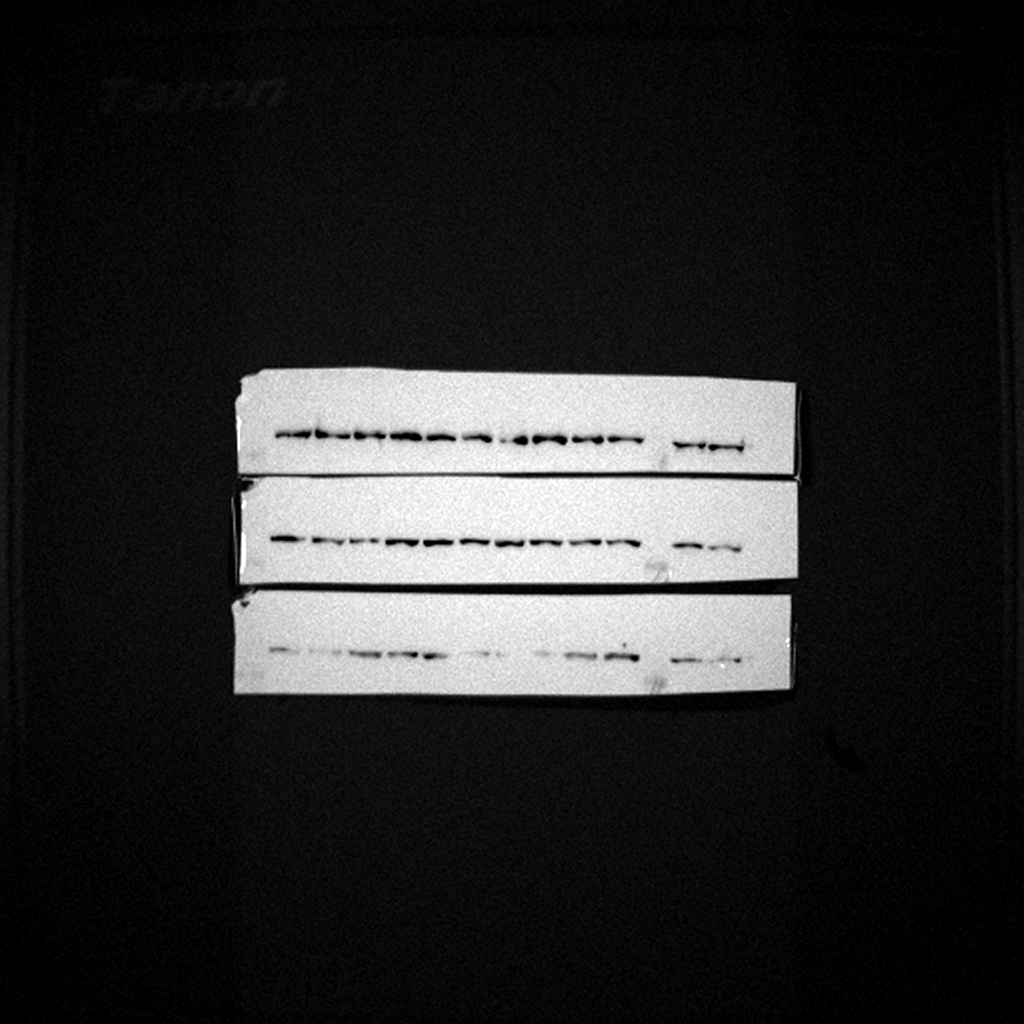

Supplement: Supplementary file 1 [file DataSheet_1.zip › 496241 original Western Blot images/origin figure/pRbs780-pRbs807811.tif]

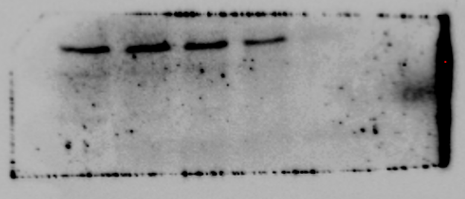

Supplement: Supplementary file 1 [file DataSheet_1.zip › 496241 original Western Blot images/origin figure/Pro-caspase3.tif]

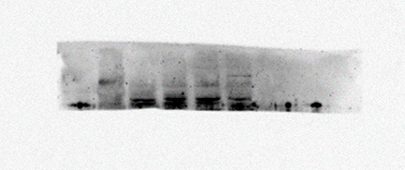

Supplement: Supplementary file 1 [file DataSheet_1.zip › 496241 original Western Blot images/origin figure/Rb.tif]

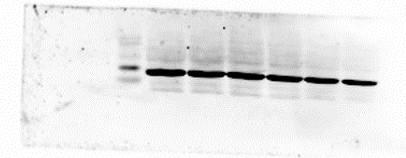

Supplement: Supplementary file 1 [file DataSheet_1.zip › 496241 original Western Blot images/origin figure/the actin of p53 and Rb.tif]

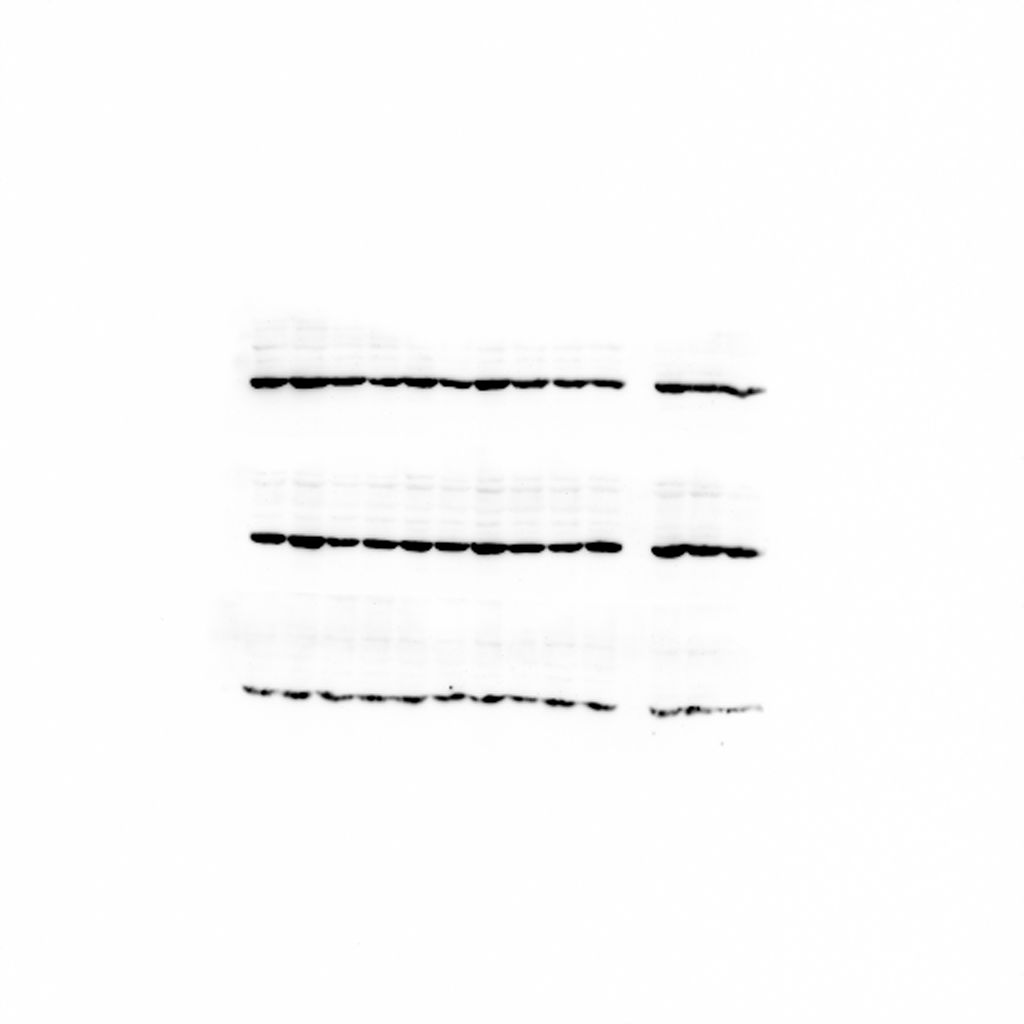

Supplement: Supplementary file 1 [file DataSheet_1.zip › 496241 original Western Blot images/origin figure/the actin of pRbs780 and pRbs807811.tif]

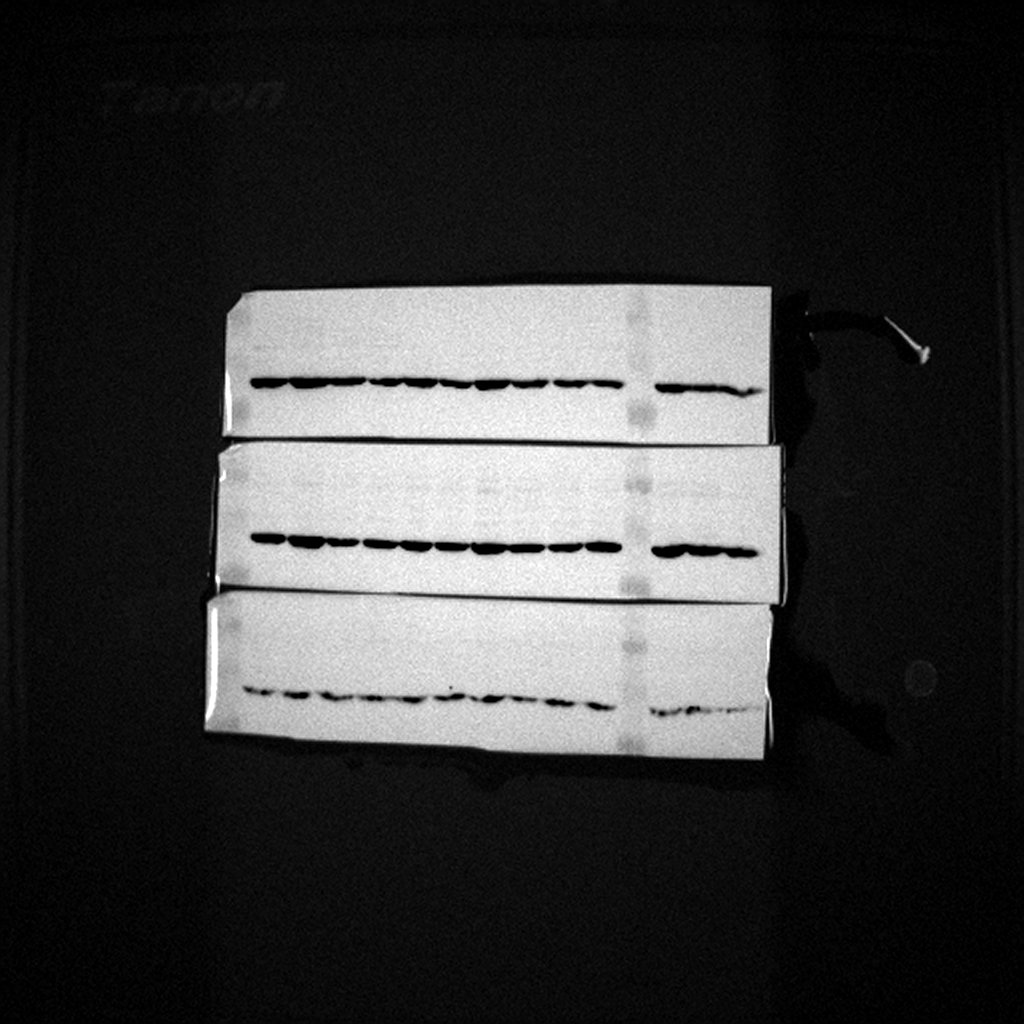

Supplement: Supplementary file 1 [file DataSheet_1.zip › 496241 original Western Blot images/origin figure/the actin of pRbs780-pRbs807811.tif]

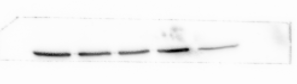

Supplement: Supplementary file 1 [file DataSheet_1.zip › 496241 original Western Blot images/origin figure/the actin of Pro-caspase3 and Bcl-2.tif]
